# Role of the multi-drug efflux systems on the baseline susceptibility to ceftazidime/avibactam and ceftolozane/tazobactam in clinical isolates of non-carbapenemase-producing carbapenem-resistant *Pseudomonas aeruginosa*

**DOI:** 10.3389/fphar.2022.1007162

**Published:** 2022-10-03

**Authors:** María José Contreras-Gómez, José R. W. Martinez, Lina Rivas, Roberto Riquelme-Neira, Juan A. Ugalde, Aniela Wozniak, Patricia García, José M. Munita, Jorge Olivares-Pacheco, Manuel Alcalde-Rico

**Affiliations:** ^1^ Grupo de Resistencia Antimicrobiana en Bacterias Patógenas y Ambientales (GRABPA), Instituto de Biología, Pontificia Universidad Católica de Valparaíso, Valparaíso, Chile; ^2^ Genomics and Resistant Microbes Group (GeRM), Instituto de Ciencias e Innovación en Medicina (ICIM), Facultad de Medicina, Clínica Alemana, Universidad Del Desarrollo, Santiago, Chile; ^3^ Millennium Initiative for Collaborative Research on Bacterial Resistance (MICROB-R), Santiago, Chile; ^4^ Núcleo de Investigaciones Aplicadas en Ciencias Veterinarias y Agronómicas, Facultad de Medicina Veterinaria y Agronomía, Universidad de Las Américas, Santiago, Chile; ^5^ Center for Bioinformatics and Integrative Biology, Facultad de Ciencias de la Vida, Universidad Andrés Bello, Santiago, Chile; ^6^ Laboratory of Microbiology, Department of Clinical Laboratories, Escuela de Medicina, Pontificia Universidad Católica de Chile, Santiago, Chile; ^7^ Clinical Laboratories Network, Red de Salud UC-CHRISTUS, Santiago, Chile

**Keywords:** ceftazidime/avibactam, cefotolozane/tazobactam, RND efflux pump, baseline susceptibility, carbapenem resistant *Pseudomonas aeruginosa*

## Abstract

Carbapenem-resistant *Pseudomonas aeruginosa* (CRPA) is one of the pathogens that urgently needs new drugs and new alternatives for its control. The primary strategy to combat this bacterium is combining treatments of beta-lactam with a beta-lactamase inhibitor. The most used combinations against *P. aeruginosa* are ceftazidime/avibactam (CZA) and ceftolozane/tazobactam (C/T). Although mechanisms leading to CZA and C/T resistance have already been described, among which are the resistance-nodulation-division (RND) efflux pumps, the role that these extrusion systems may play in CZA, and C/T baseline susceptibility of clinical isolates remains unknown. For this purpose, 161 isolates of non-carbapenemase-producing (Non-CP) CRPA were selected, and susceptibility tests to CZA and C/T were performed in the presence and absence of the RND efflux pumps inhibitor, Phenylalanine-arginine β-naphthylamide (PAβN). In the absence of PAβN, C/T showed markedly higher activity against Non-CP-CRPA isolates than observed for CZA. These results were even more evident in isolates classified as extremely-drug resistant (XDR) or with difficult-to-treat resistance (DTR), where CZA decreased its activity up to 55.2% and 20.0%, respectively, whereas C/T did it up to 82.8% (XDR), and 73.3% (DTR). The presence of PAβN showed an increase in both CZA (37.6%) and C/T (44.6%) activity, and 25.5% of Non-CP-CRPA isolates increased their susceptibility to these two combined antibiotics. However, statistical analysis showed that only the C/T susceptibility of Non-CP-CRPA isolates was significantly increased. Although the contribution of RND activity to CZA and C/T baseline susceptibility was generally low (two-fold decrease of minimal inhibitory concentrations [MIC]), a more evident contribution was observed in a non-minor proportion of the Non-CP-CRPA isolates affected by PAβN [CZA: 25.4% (15/59); C/T: 30% (21/70)]. These isolates presented significantly higher MIC values for C/T. Therefore, we conclude that RND efflux pumps are participating in the phenomenon of baseline susceptibility to CZA and, even more, to C/T. However, the genomic diversity of clinical isolates is so great that deeper analyzes are necessary to determine which elements are directly involved in this phenomenon.

## Introduction

Carbapenem-resistant *Pseudomonas aeruginosa* (CRPA) has shown a substantial increase in prevalence as a nosocomial pathogen over the last years (Walkty et al., 2017; Sader et al., 2018; Qin et al., 2022; Lodise et al., 2022; Torrens et al., 2022). Until a few years ago, carbapenems were highly successful in treating multi-drug resistant (MDR) isolates of *P. aeruginosa*, but the rapid dissemination of highly efficient resistant mechanisms such as carbapenemases put the effectiveness of these drugs at risk (Labarca et al., 2016; Botelho et al., 2019; Horcajada et al., 2019; Qin et al., 2022). The main problem is that patients infected with CRPA usually have the worst clinical outcomes and a high mortality rate between 20% and 30% (Pena et al., 2012; Lodise et al., 2022). This led the World Health Organization (WHO) to grant this bacterium the category of critical priority pathogen in 2017, highlighting the requirement for new treatment options (WHO, 2017; Tacconelli et al., 2018). Fortunately, several new drugs have been recently introduced as successful options, two of which are ceftazidime/avibactam (CZA) and ceftolozane/tazobactam (C/T), both combinations of a potent cephalosporin (ceftazidime and ceftolozane) and beta-lactamase inhibitor (avibactam and tazobactam) (Goodlet et al., 2016; van Duin and Bonomo, 2016). However, due to the limited spectrum of avibactam and tazobactam inhibition, the use of CZA and C/T is mainly recommended to treat strains that do not produce metallo-beta-lactamases (i.e., VIM, NDM, SPM, or IMP), and in the specific case of C/T, neither to strains that produce class A carbapenemases (i.e., KPC) (Vasoo et al., 2015; Goodlet et al., 2016; van Duin and Bonomo, 2016), thus limiting their effectiveness to non-carbapenemase-producing CRPA (Non-CP-CRPA) isolates.

Despite CZA and C/T presenting a high activity rate and good coverage against *P. aeruginosa* clinical isolates, including MDR and CRPA strains (Goodlet et al., 2016; Pfaller et al., 2017; Pfaller et al., 2018; Horcajada et al., 2019; Sader et al., 2021), some acquired resistance mechanisms to these antibiotic combinations have been observed. It has been reported that resistance to both drugs may be developed through mutation of penicillin-binding proteins (PBPs) (Castanheira et al., 2019; Fournier et al., 2021); by horizontally-transferred beta-lactamases (Ortiz de la Rosa et al., 2019; Arca-Suarez et al., 2021; Fraile-Ribot et al., 2021; Teo et al., 2021; Poirel et al., 2022; Ruedas-Lopez et al., 2022); or by overexpression and/or structural modifications in the Ω-loop region of the chromosomally-encoded beta-lactamase, AmpC (Cabot et al., 2014; Fraile-Ribot et al., 2017; Cabot et al., 2018; Chalhoub et al., 2018; Arca-Suarez et al., 2020; Compain et al., 2020; Teo et al., 2021; Ruedas-Lopez et al., 2022). In addition, mutations leading to overexpression and/or structural modification of the resistance-nodulation-division (RND) efflux system, MexAB-OprM, are associated with resistance to CZA (Chalhoub et al., 2018; Sanz-Garcia et al., 2018; Castanheira et al., 2019). Regarding C/T, studies support a higher affinity of ceftolozane for certain PBPs than other beta-lactams, and C/T activity is less affected by changes in efflux pumps and porin permeability, hindering the emergence of C/T resistance mechanisms (Cabot et al., 2014; Wi et al., 2018; Martin-Loeches et al., 2020). However, over-expression of MexAB-OprM and/or structural modification of MexCD-OprJ have been directly implicated in decreased susceptibility to C/T (Castanheira et al., 2021; Gomis-Font et al., 2021).

Although both the overexpression and mutations of RND efflux pumps are considered acquired resistance mechanisms, some of these systems actively participate in the intrinsic resistance of *P. aeruginosa* (Alvarez-Ortega et al., 2013; Hernando-Amado et al., 2016). The concept of intrinsic resistance is highly applicable to the study of reference strains (e.g., PAO1 and PA14), but it is not enough to explain the phenomena observed in clinical isolates. This is mainly because the ancestral origin of these strains and their historical exposition to multiple and variable antimicrobials are often unknown. Based on that, we propose the concept of “baseline susceptibility” since it is interesting to deal with aspects of intrinsic resistance that cannot be ascribed to this definition in clinical isolates. Therefore, the concept of “baseline susceptibility” could be understood as the level of susceptibility that a particular group of clinical isolates present to a given antibiotic, irrespective of their previous exposure to antimicrobial agents. Even though the potential effect of acquired mutations over RND-mediated resistance to CZA and C/T has been previously explored, the role that RND efflux systems play in the baseline susceptibility to CZA and C/T remains to be defined.

The classical methodology to study the intrinsic resistance mechanisms is based on antibiotic susceptibility tests carried out in reference strains and their isogenic loss-of-function mutants (Alvarez-Ortega et al., 2011; Fernandez et al., 2013; Chalhoub et al., 2017; Sanz-Garcia et al., 2019). Due to the limitations of this methodology in clinical isolates, alternative strategies should be addressed to evaluate the mechanisms implicated in the baseline susceptibility to antibiotics in these strains. Regarding the RND systems, efflux pump inhibitors (EPIs) are particularly relevant because the combined use of EPIs and antibiotics is being explored as a promising adjuvant strategy to overcome MDR- and virulence-mediated by RND efflux systems (Adamson et al., 2015; Wright, 2016; Blanco et al., 2018; Gbian and Omri, 2021). Phenylalanine-arginine β-naphthylamide (PAβN) is the most active and best-studied inhibitor of RND efflux systems, broadly used to explore the role of these genetic determinants in the antibiotic resistance (Kriengkauykiat et al., 2005; Tohidpour et al., 2009; Fuste et al., 2013; Chalhoub et al., 2016; Shields et al., 2022) and virulence (Hirakata et al., 2009; Adamson et al., 2015; El-Shaer et al., 2016; Rampioni et al., 2017; Li et al., 2022) in *P. aeruginosa* and other bacterial species (Dupont et al., 2016; Castanheira et al., 2020). This peptidomimetic compound inhibits the RND efflux activity through competition with antibiotics for binding and subsequent extrusion through RND efflux systems, decreasing the efficiency of RND-mediated efflux of antibiotics (Askoura et al., 2011; Fujiwara et al., 2022). Considering all the mentioned evidence, the main objectives of this study are to determine the susceptibility to CZA and C/T in Non-CP-CRPA clinical isolates collected from 12 Chilean hospitals and, using PAβN as an efflux pump inhibitor, to assess the potential role that RND systems may play in their baseline susceptibility to these new combined treatments (WHO, 2017).

## Materials and methods

### Strain collection

Clinical isolates of CRPA were collected from 12 Chilean hospitals from December 2018 to December 2020 in association with the *Millennium Initiative for Collaborative Research on Bacterial Resistance*, MICROB-R (www.microb-r.org). Isolates were sent to a central laboratory at the Genomics and Microbial Resistance laboratory (GeRM) at Universidad del Desarrollo (Santiago, Chile) for further analysis. Species identification was confirmed by MALDI-TOF (Vitek MS bioMérieux, Durham, NC), and carbapenem resistance was evaluated by disk diffusion method (see below). Isolates showing resistance to imipenem (IMI), meropenem (MEM), or both were selected, obtaining 243 CRPA. Finally, 82 isolates were discarded due to the presence of carbapenemases (see below), thus bringing a total of 161 Non-CP-CRPA, some of which were also included in a previously published study in 2021 (Rivas et al., 2021).

### Carbapenemase detection assays

To select non-carbapenemase-producing strains, phenotypic tests for carbapenemase activity (BlueCarba) and molecular tests (polymerase chain reaction, PCR) for detection of *bla*_VIM_, *bla*_KPC_, *bla*_NDM_, *bla*_SPM,_ or *bla*_IMP_ genes were performed. The BlueCarba assay was carried out following the protocol previously reported (Pires et al., 2013). The molecular detection of the carbapenemase genes was carried out in two parallel multiplex PCR using the paired primers and conditions listed in Table 1 (Ellington et al., 2007; Wolter et al., 2009; Poirel et al., 2011). The GoTaq G2 Green Master Mix PCR (Promega) was used for PCR reaction following the manufacturer’s guidelines. DNA was extracted from two or three colonies resuspended in 75 µl of ultrapure distilled water (Invitrogen™) by heat lysis (95°C for 10 min), and 1 µl was used as DNA template (Ejaz et al., 2020). Finally, all isolates that showed evidence of carrying carbapenemases by either or both of these techniques were considered carbapenemase producers, being thus excluded from this study.

**TABLE 1:** Conditions and sequence of the primers used in the Multiplex PCR for molecular detection of the carbapenemase genes *bla*_VIM_, *bla*_KPC_, *bla*_NDM_, *bla*_SPM,_ or *bla*_IMP_.

PCR reaction	Carbapenemase gene	Sequence (5′→3′)	Product size (bp)	Annealing temperature	References
Multiplex 1	*bla*_KPC_	CGT​CTA​GTT​CTG​CTG​TCT​TG	798	55°C	Poirel et al., 2011
		CTT​GTC​ATC​CTT​GTT​AGG​CG			
	*bla*_VIM_	GGT​GTT​TGG​TCG​CAT​ATC​GC	504		Wolter et al., 2009
		CCA​TTC​AGC​CAG​ATC​GGC​ATC			
	*bla*_IMP_	GGAATAGAGTGGCTTAAYTCTC	188		Ellington et al., 2007
		CCAAACYACTASGTTATCT			
Multiplex 2	*bla*_NDM_	GGT​TTG​GCG​ATC​TGG​TTT​TC	452	63°C	Poirel et al., 2011
		CGG​TGA​TAT​TGT​CAC​TGG​TGT​GG			
	*bla*_SPM_	AAA​ATC​TGG​GTA​CGC​AAA​CG	271		Ellington et al., 2007
		ACA​TTA​TCC​GCT​GGA​ACA​GG			

### Disk diffusion test

Susceptibility to imipenem 10 µg (IPM), meropenem 10 µg (MEM), aztreonam 30 µg (ATM), ceftazidime 30 µg (CAZ), cefepime 30 µg (FEP), piperacillin/tazobactam 100/10 µg (TZP), ciprofloxacin 5 µg (CIP), amikacin 30 µg (AK) and gentamicin 10 µg (CN) was determined through the disk-diffusion method following CLSI 2022 recommendations (CLSI, 2020) (Supplementary Table S1); we used Mueller Hinton agar II BBL^TM^ (Becton, Dickinson; BD) and Oxoid^TM^ disk. Quality control (QC) tests were performed using the reference strain *P. aeruginosa* ATCC 27853, and all QC were within the CLSI acceptable range. The phenotypes “susceptible”, “intermediate” and “resistant” were defined for each antibiotic based on their respective zone diameter breakpoints established by CLSI (CLSI, 2020). The “non-susceptible” group included the “intermediate” and “resistant” categories. Then, the isolates were classified into four independent groups based on their multi-drug resistance profile (Magiorakos et al., 2012; Kadri et al., 2018; Giannella et al., 2019), as follows: 1) difficult-to-treat resistance (DTR), including isolates that were non-susceptible to all beta-lactams and quinolones used in this study; 2) extensively drug-resistant (XDR), including isolates non-susceptible to five antibiotic families or more, and not considered DTR; 3) MDR, non-DTR and non-XDR isolates presenting a non-susceptible phenotype to three or four antibiotic families; 4) Non-MDR, isolates not included in any of the above categories.

### Broth microdilution assay

The minimum inhibitory concentration (MIC) of ATM, CIP, CZA, and C/T were determined through broth microdilution (BMD) assay following CLSI M100 guidelines (CLSI, 2020). Cation-adjusted Mueller-Hinton broth (MHB-II) (BBL^TM^ BD, Sparks, MD) and 96-well round-bottom plates were used (Thermo Scientific™). All antimicrobial compounds were obtained from Merck, except CAZ and PAβN, which were obtained from Sigma-Aldrich, and avibactam, which was donated by Pfizer, Inc. Concentrations spanned the doubling dilution range of 0.125 μg/ml to 64 μg/ml for ceftazidime and ceftolozane, whereas the concentration of avibactam and tazobactam were kept at 4 μg/ml. The double dilution range analyzed for ATM and CIP concentrations were 0.25–128 μg/ml and 0.008–4 μg/ml, respectively. MIC values were determined after incubation for 18–20 h at 37°C, and three or more biological replicates were performed. As a growth control, strains were inoculated in wells containing MHB-II or MHB-II + PAβN without CZA and C/T. As sterile control, bacteria were not inoculated in wells containing MHB-II or MHB-II + PAβN without CZA and C/T. QC tests were performed using the reference strain *P. aeruginosa* ATCC 27853, and all QC were within the CLSI acceptable range. The phenotypes “susceptible”, “intermediate” and “resistant” were defined for CZA and C/T based on CLSI breakpoints (CLSI, 2020). In the case of C/T, the “non-susceptible” group included the “intermediate” and “resistant” categories. To evaluate the effect of RND efflux pump inhibition on ATM, CIP, CZA, and C/T susceptibility, BMD was performed as described above, but in the presence of 25 μg/ml of PAβN (Askoura et al., 2011; Blanco et al., 2018). It was considered that the RND efflux activity contributed to the baseline susceptibility to CZA and/or C/T when a two-fold (named “RND 2x”) or more (named “RND ≥4x”) decrease in MIC values were observed in the presence of PAβN (overall named “RND” isolates). On the contrary, isolates with equal or higher CZA and/or C/T MIC values in the presence of PAβN were classified as “Non-RND”. Four of the 161 Non-CP-CRPA clinical isolates were excluded due to the lack of growth observed in the presence of PAβN.

### Statistical test

To evaluate the type and significance of the correlation between CZA and C/T MIC distribution in Non-CP-CRPA clinical isolates, we used the Spearman’s rank correlation test. To determine whether Non-CP-CRPA showed a significant variation of overall CZA or C/T susceptibility in the presence of PAβN, we performed the Wilcoxon matched-pairs signed rank test. Normal distribution (parametric) of the CZA or C/T MIC values was assessed with D'Agostino and Pearson, Shapiro-Wilk, and Kolmogorov-Smirnov tests. Once determined that the samples do not follow a parametric distribution, we performed a Kruskal–Wallis test to analyze the significance of the CZA or C/T MIC variations observed in isolates classified as Non-RND, RND 2x, and RND ≥4x. All analyses were performed using GraphPad Prism 9.0 (San Diego, CA. United States). Significant differences were considered when *p*-*value* was <0.05 (*), *p* < 0.01 (**), *p* < 0.005 (***) and *p* < 0.001 (****).

## Results

### Identification and characterization of non-CP-CRPA clinical isolates

This study included 161 Non-CP-CRPA clinical isolates, selected among 243 CRPA collected from December 2018 to December 2020. These Non-CP-CRPA isolates showed resistance to imipenem and/or meropenem, but no enzyme activity or molecular evidence of carbapenemase genes was detected (Supplementary Table S1). The source of these isolates was tissue samples (*n* = 77; 47.8%), blood (*n* = 51; 31.7%), other sterile fluids (*n* = 17; 10.6%), respiratory tract (*n* = 11; 6.8%) and urine (*n* = 5; 3.1%). The isolates were classified into four independent groups according to their multi-drug resistance profile (Non-MDR, MDR, XDR, and DTR) (Magiorakos et al., 2012; Kadri et al., 2018; Giannella et al., 2019). The results (Table 2) showed that 53 isolates (32.9%) had a non-MDR phenotype, 49 (30.5%) were considered MDR, 29 (18%) XDR, and 30 (18.6%) were grouped into the category DTR. These results evidence the high level of collateral antibiotic resistance (other than carbapenems) associated with the CR phenotype, with over 67% of Non-CP-CRPA isolates included in the MDR, XDR, and DTR categories.

**TABLE 2:** **CZA and C/T susceptibility profile among Non-CP-CRPA clinical isolates with different multi-drug resistance phenotypes**.

	N^o^ isolates (%)	MIC CZA (µg/ml)				MIC C/T (µg/ml)				
		MIC_50_	MIC_90_	S (%)	R (%)	MIC_50_	MIC_90_	S (%)	I (%)	R (%)
Non-MDR	53 (32.9)	4	8	49 (92.5)	4 (7.5)	1	1	52 (98.1)	1 (1.9)	0 (0.0)
MDR	49 (30.5)	8	16	37 (75.5)	12 (24.5)	1	4	45 (91.8)	3 (6.1)	1 (2.0)
XDR	29 (18)	8	64	16 (55.2)	13 (44.8)	2	32	24 (82.8)	1 (3.4)	4 (13.8)
DTR	30 (18.6)	16	32	6 (20.0)	24 (80.0)	2	32	22 (73.3)	4 (13.3)	4 (13.3)
Total	161 (100)	8	32	108 (67.1)	53 (32.9)	1	8	143 (88.8)	9 (5.6)	9 (5.6)

MIC: minimum inhibitory concentration; CZA: ceftazidime/avibactam; C/T: ceftolozane/tazobactam; S: susceptible; I: intermediate; R: resistant; Non-MDR: non multi-drug resistant; MDR: multi-drug resistant; XDR: extremely drug resistant; DTR: difficult-to-treat resistance.

### The activity of CZA strongly decreases in non-CP-CRPA isolates with an XDR or DTR phenotype

**FIGURE 1:**
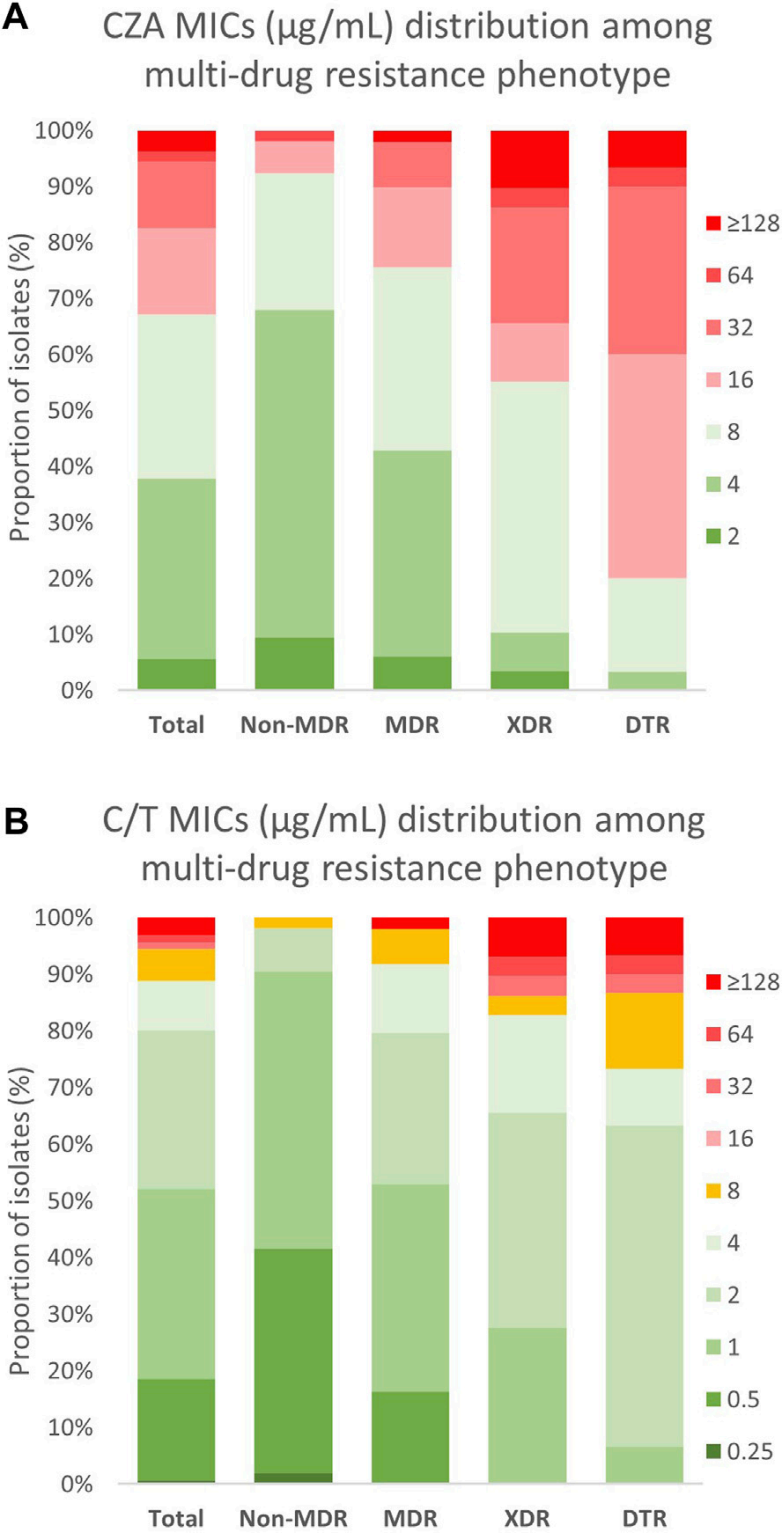
MIC distribution of CZA and C/T among different multi-drug resistance phenotypes in Non-CP-CRPA clinical isolates. Non-CP-CRPA isolates (Total) were classified as Non-MDR, MDR, XDR, and DTR. The distribution of **(A)** CZA and **(B)** C/T MIC values among these groups are represented in the graphic. Green, yellow and red colors indicate MIC values considered by CLSI as “susceptible”, “intermediate”, and “resistant”, respectively. It is observed that acquisition of each more multi-drug resistance phenotype is strongly associated with the increase of **(A)** CZA and **(B)** C/T MIC values, in particular for CZA.

Susceptibility to CZA was determined by broth microdilution (BMD) following CLSI guidelines (CLSI, 2020). The results showed that, although 67.1% (*n* = 108) of Non-CP-CRPA isolates remain susceptible to CZA (MIC ≤8 μg/ml) (Table 2), most of them presented MIC values close to the clinical breakpoint (Figure 1A), reason by which high MIC_50_ and MIC_90_ values (8 and 32 μg/ml, respectively) were observed (Table 2). To evaluate how the multi-drug resistance profile affects CZA susceptibility, we analyzed the MIC distribution of CZA in those isolates classified as Non-MDR, MDR, XDR, and DTR (Figure 1A). We found that both Non-MDR and MDR isolates presented a reasonable susceptibility rate to CZA (92.5% and 75.5%, respectively). Still, in XDR and DTR isolates, which showed a high multi-drug resistance rate, the susceptibility to CZA strongly decreased to 55.2% and 20%, respectively (Table 2 and Figure 1A). This is also observable with MIC_50/90_ values (Table 2), spanning from 4/8 and 8/16 μg/ml in Non-MDR and MDR isolates to 8/64, and 16/32 μg/ml in XDR and DTR isolates, respectively. Altogether, these results evidence that although most of the Non-CP-CRPA isolates remain susceptible to CZA (67.1%), they present a low susceptibility rate with MIC values close to or higher than the clinical breakpoint. Furthermore, acquiring a high multi-drug resistance rate is a critical factor that negatively impacts CZA activity.

### C/T is highly active against non-CP-CRPA, even in isolates with an XDR or DTR phenotype

Similarly to CZA, the determination of C/T susceptibility in Non-CP-CRPA was carried out through the BMD method. The overall susceptibility was 88.8% (*n* = 143) and most of the isolates classified as “susceptible” (90.2%) presented MIC values between 0.5–2 μg/ml, which is at least 4-fold lower than the “intermediate” breakpoint (8 μg/ml) (Table 2 and Figure 1B). The MIC_50/90_ values were 1/8 μg/ml, substantially lower than those obtained for CZA (8/32 μg/ml). The MIC distribution (Figure 1B) and MIC_50/90_ values (Table 2) obtained for each multi-drug resistance category (Non-MDR, MDR, XDR, and DTR) showed that like what happened with CZA, those isolates with a high multi-drug resistance profile (XDR or DTR) exhibited lower susceptibility to C/T. However, the impact of an XDR or DTR phenotype on C/T resistance was considerably lower than that observed in CZA since the susceptibility observed was 98.1% (Non-MDR), 91.8% (MDR), 82.8% (XDR), and 73.3% (DTR) (Table 2). This indicates that C/T shows high activity against Non-CP-CRPA isolates, even in isolates with an XDR or DTR phenotype, suggesting that C/T could be a successful treatment against Non-CP-CRPA with a high multi-drug resistance rate.

### Clinical resistance to C/T is strongly associated with a CZA-resistant phenotype in non-CP-CRPA, but not vice versa.

**FIGURE 2:**
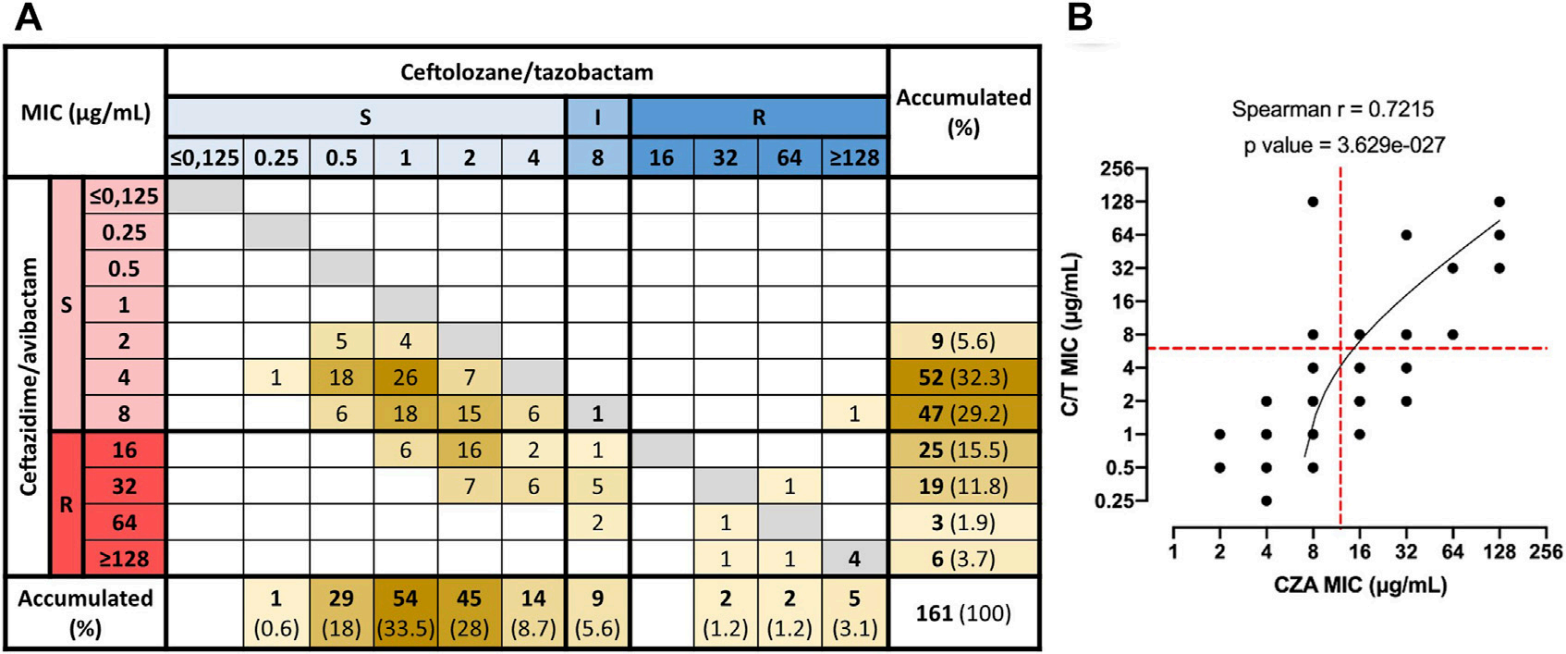
Correlation between MIC values of CZA and C/T in Non-CP-CRPA clinical isolates. Combinations of CZA and C/T MIC values for each one of the Non-CP-CRPA isolates were quantified and represented in a matrix **(A)**. MIC values were grouped as S (susceptible), I (intermediate), and R (resistant) according to the breakpoint of CZA and C/T established by CLSI (2020). **(B)** The correlation analysis (Spearman’s rank correlation test) showed that the MIC distribution of CZA and C/T presented a significant positive correlation (Spearman r: 0.7215; *p* < 0.0001). However, most of the isolates (95.7%) had lower C/T MIC values than those obtained for CZA, indicating that C/T presented a higher activity than CZA overall Non-CP-CRPA isolates. Furthermore, most CZA-resistant isolates remained susceptible to C/T, but most C/T-resistant isolates also showed a CZA-resistant phenotype.

To evaluate the relationship between CZA and C/T susceptibility in individual Non-CP-CRPA isolates, we generated a matrix in which the abundance of the different combinations of CZA and C/T MIC values were quantified (Figure 2A). The results showed a significant positive correlation (Spearman r: 0.7215; *p* < 0.0001) between the susceptibility to both antibiotics, meaning that: as the susceptibility to CZA or C/T decreases, susceptibility to the other also declines. However, most of the isolates (95.7%) presented lower MIC values of C/T than those observed in CZA (Figure 2A and Table 2). For that reason, 98.2% of the isolates susceptible to CZA (*n* = 108) were also susceptible to C/T (*n* = 106), and a high proportion of CZA resistant isolates remained susceptible to C/T (69.8%; 37/53) or with an intermediate phenotype (15.1%; 8/53) (Figure 2B). On the contrary, 88.9% of the Non-CP-CRPA with a non-susceptible phenotype to C/T were also CZA resistant. Moreover, the only two C/T resistant isolates that remained susceptible to CZA presented the highest MIC value accepted for CZA susceptible phenotype (8 μg/ml) (Figure 2A). These results show evidence that C/T remains active in a high proportion of Non-CP-CRPA with a CZA-resistant phenotype. In addition, a C/T resistant phenotype is strongly associated with CZA resistance in most of the Non-CP-CRPA isolates.

### The inhibition of RND efflux pumps by PAβN improves the susceptibility to CZA and C/T in a high proportion of non-CP-CRPA clinical isolates

**FIGURE 3:**
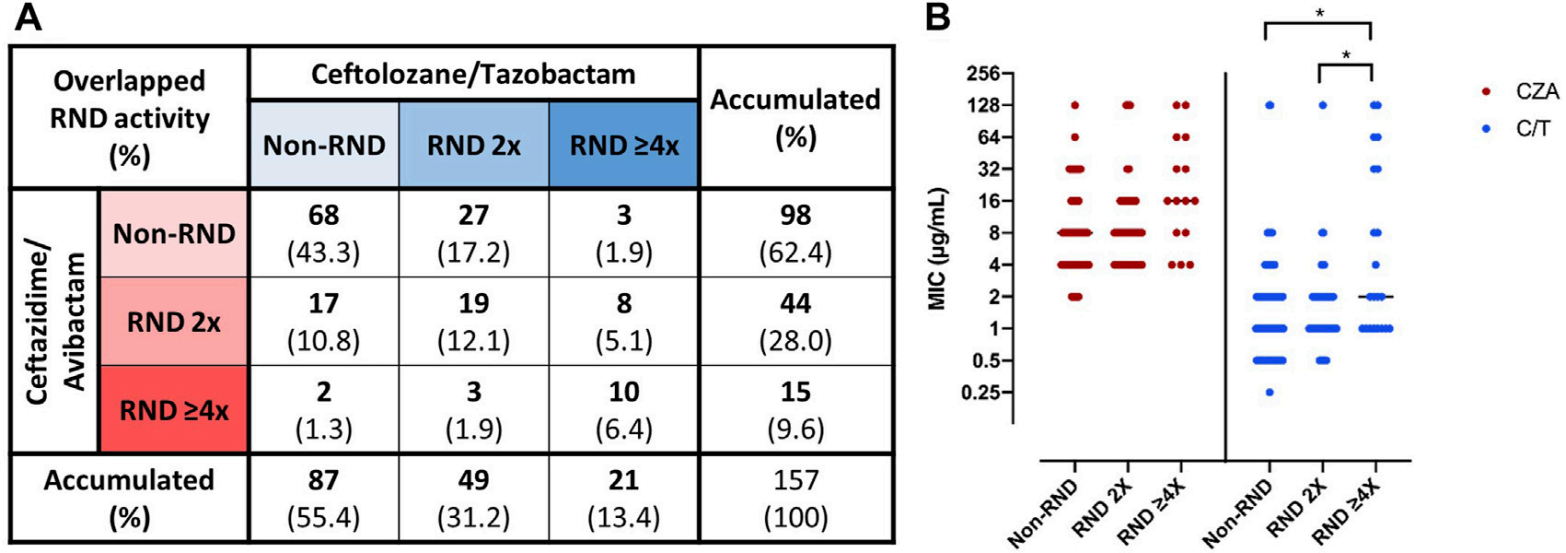
Role of RND efflux systems on “baseline susceptibility” to CZA and C/T in Non-CP-CRPA clinical isolates. Non-CP-CRPA isolates were classified as “Non-RND”, “RND 2x”, and “RND ≥4x” according to the increase in the levels of CZA or C/T susceptibility in the presence of the efflux pump inhibitor, PAβN. The isolates with equal or higher MIC values in the presence of PAβN were named “Non-RND”, and no contribution of the RND efflux systems in CZA or C/T susceptibility was considered. The isolates whose MIC values decreased two-fold or more than two-fold in the presence of PAβN were named “RND 2x” or “RND ≥4x”, respectively, and a role of the RND efflux systems in CZA or C/T susceptibility was considered. Panel **(A)** shows the proportion of the Non-CP-CRPA isolates with the different combinations of overlapped RND contribution to CZA and/or C/T susceptibility. Panel **(B)** shows the CZA and C/T MIC distribution among the individual isolates classified as “Non-RND”, “RND 2x”, and “RND ≥4x”. The results showed that **(A)** 37.6% (*n* = 59), 44.6% (*n* = 70), and 25.5% (*n* = 40) of Non-CP-CRPA analyzed (*n* = 157) were considered as “RND” (sum of “RND 2x” and “RND ≥4x”) for CZA, C/T, or both, respectively. The CZA and C/T MIC distribution among “Non-RND”, “RND 2x” and “RND ≥4x” isolates showed a significant increase of C/T MIC values in “RND ≥4x”, evidencing that RND may contribute to a high level of C/T resistance.

To assess the role of RND efflux systems in the baseline susceptibility to CZA and C/T of Non-CP-CRPA clinical isolates, we determined the MIC of these two new drug combinations in the presence of the efflux pump inhibitor, PAβN. To explore the cut-off point at which increased susceptibility to a specific antibiotic may be associated with inhibition of RND activity, MIC determination of CIP and ATM, two known antibiotics that are extruded by the constitutively expressed MexAB-OprM efflux system, in the presence and absence of PAβN were carried out in three reference strains (PAO1, PA14 and ATCC 27853). The results showed that MIC to ATM, an antibiotic exclusively extruded by MexAB-OprM, decreased two-fold in two out of three strains analyzed in the presence of PAβN (Supplementary Table S2). In the same way, the results showed that MIC to CIP, an antibiotic that several efflux systems may extrude (mainly MexAB-OprM, MexCD-OprJ, and MexEF-OprN), decreased two- or four-fold in all strains exposed to PAβN. Therefore, the isolates were classified as Non-RND, RND 2x, and RND ≥4x according to the estimated contribution of the RND efflux systems to CZA or C/T baseline susceptibility, as described in the methods section. Globally, the Wilcoxon matched pairs signed rank test showed that the presence of PAβN led to a significant reduction in overall susceptibility to C/T (*p* = 0.0003) but not to CZA (*p* = 0.1011). However, 37.6% (59/157) and 44.6% (70/157) of Non-CP-CRPA analyzed were considered as “RND” for CZA or C/T, respectively, whereas 25.5% (40/157) were considered as “RND” for both CZA and C/T (Figure 3A). Moreover, 25.4% (15/59) and 30% (21/70) of “RND” isolates increased more than two-fold (RND ≥4x) their CZA or C/T susceptibility, respectively (Figure 3A). Comparing the MIC values obtained for CZA or C/T among the different groups Non-RND, RND 2x, and RND ≥4x (Figure 3B), it was observed that the RND ≥4x isolates exhibited significantly higher MIC values of C/T than those obtained in Non-RND and RND 2x isolates (*p* = 0.0159 and 0.0425, respectively). Moreover, a higher proportion of “RND” phenotype in CZA and C/T non-susceptible isolates was observed compared with those susceptible, with an increase of 32.7%–48% for CZA and 42.5%–61.1% for C/T.

## Discussion

It is generally assumed that clinical isolates present similar intrinsic resistance mechanisms to those described in reference strains. Notwithstanding, based on the genomic diversity of clinical isolates, it is possible that many of these mechanisms do not have the same contribution to the intrinsic resistance in all bacterial isolates (Nonaka et al., 2010; Darch et al., 2015; Varin et al., 2017). In addition, it is difficult to know whether or not a clinical isolate has been previously exposed to a specific antibiotic since these pathogens can cause intrahospital outbreaks, making it difficult to monitor the overall antibiotic exposure of one specific clinical strain (Chaves et al., 2017; Buhl et al., 2019; Mihara et al., 2020), and can survive in hospital environments (Varin et al., 2017), where they are intensively exposed to disinfectants and other antimicrobial compounds that may co-select antibiotic resistance mechanisms (Chuanchuen et al., 2001; Hernandez et al., 2011). These highlights the need to apply a modified intrinsic resistance concept that can be advantageous when clinical isolates are studied, named here as “baseline susceptibility”. It has been reported that modification and/or overexpression of the RND efflux pumps are implicated in acquired resistance to CZA and C/T in Non-CP-CRPA, usually in combination with other mechanisms of beta-lactam resistance such as the *oprD* decrease expression, PBPs modification or *ampC* overexpression (Castanheira et al., 2014; Wi et al., 2018; Castanheira et al., 2021; Gomis-Font et al., 2021). In particular, the overexpression and/or structural modification of MexAB-OprM is the most common RND-mediated mechanism observed in acquired resistance to CZA and C/T (Castanheira et al., 2014; Wi et al., 2018; Gomis-Font et al., 2021). This efflux system is usually constitutively expressed in *P. aeruginosa* reference strains (PAO1, PA14), and their role in the intrinsic resistance to a large variety of antibiotics has been broadly reported (Morita et al., 2001; Hernando-Amado et al., 2016). In this study the role of efflux pumps in the baseline susceptibility to CZA and C/T of Non-CP-CRPA clinical isolates was addressed.

The results showed that a large percentage of strains decreased their MIC to CZA and C/T in the presence of the efflux pump inhibitor, PAβN, but most of them were minor changes (RND 2x) and insufficient to increase the proportion of susceptible strains in this population. This may indicate that the effectiveness of the RND-mediated efflux of CZA and C/T is generally low compared with other antibiotics, consistent with previous reports (Cabot et al., 2014; Castanheira et al., 2014; Castanheira et al., 2021). In fact, the changes observed at the population scale in susceptibility to CZA due to the presence of PAβN were not significant, opposite to C/T, for which these changes were significant. However, there was a non-minor proportion of these isolates in which RND efflux activity highly contributed to their baseline susceptibility (RND ≥4x). Interestingly, the RND ≥4x isolates proportionally presented higher C/T MIC values than RND 2x and non-RND isolates, suggesting that a more significant contribution of RND-mediated baseline susceptibility to C/T is associated with high-level of C/T MIC in Non-CP-CRPA. These results show that efflux inhibition affects C/T susceptibility to a greater extent than CZA susceptibility. At the single strain level, the efflux pump inhibition differentially increased the susceptibility to CZA, C/T, or both, in 12.1%, 19.1%, and 25.5% of Non-CP-CRPA isolates, respectively, evidencing distinct patterns of RND-mediated contribution to the baseline susceptibility. Despite one of the limitations of this study is the lack of direct evidence about what specific RND efflux systems are implicated in the baseline susceptibility to one or both combined antibiotics, several studies have shown that MexAB-OprM, one of the central RND efflux systems implicated in intrinsic resistance (Morita et al., 2001; Hernando-Amado et al., 2016), and MexCD-OprJ are associated with acquired resistance to CZA and C/T (Chalhoub et al., 2018; Sanz-Garcia et al., 2018; Castanheira et al., 2019; Castanheira et al., 2021; Gomis-Font et al., 2021). Taking this into account, it would be possible that different combinations of their expression level and variations in their gene sequences could be underlying in the different patterns observed. However, further analysis may be performed to evaluate this hypothesis in more detail, and the role of other RND efflux systems should not be discarded.

Another contribution of this study is that Non-CP-CRPA isolates presented high levels of susceptibility to CZA and C/T, consistent with previously published studies in which both combined treatments are suggested to be effective against CRPA (Grupper et al., 2017; Shortridge et al., 2018; Wi et al., 2018; Sader et al., 2020; Lomovskaya et al., 2021; Rivas et al., 2021; Cruz-Lopez et al., 2022). Interestingly, our results showed that CZA and C/T activity decrease considerably as strains exhibited a wider multi-drug resistance profile, especially in XDR and DTR strains. Similar evidence was also reported in a multicenter study conducted in the U.S. (Grupper et al., 2017), showing that strains with resistance to all beta-lactam analyzed (FEP, CAZ, TZP, and ATM) presented a decrease in both CZA and C/T susceptibility with respect overall CRPA, being this decrease substantially higher in the case of CZA. This agreement between our results and those reported by Grupper *et al.* (Grupper et al., 2017) could be partially explained by the increased repertoire of beta-lactam resistance mechanisms in our XDR (all of them with CR phenotype) and DTR (resistant to all the beta-lactams and quinolones analyzed) isolates. Indeed, it has been shown that mechanisms usually associated with beta-lactam and carbapenem resistance (Moya et al., 2012; Castanheira et al., 2014; Cabot et al., 2018; Ruedas-Lopez et al., 2022) are also implicated in resistance to CZA (Chalhoub et al., 2018; Sanz-Garcia et al., 2018; Castanheira et al., 2019) and, to a lesser extent, to C/T (Cabot et al., 2014; Wi et al., 2018; Fournier et al., 2021; Ruedas-Lopez et al., 2022). Accordingly, the high difference here reported between CZA and C/T activity in DTR isolates, a category that have not been previously considered in this kind of studies (Del Barrio-Tofino et al., 2017; Grupper et al., 2017; Pfaller et al., 2017; Wi et al., 2018; Sader et al., 2021; Torrens et al., 2022), evidencing that the effectiveness of CZA treatment could be compromised in this group of strains. Otherwise, the high rate of C/T activity, even in CR-XDR (82.8%) and DTR (73.3%) groups, reinforces that C/T is less affected by mechanisms of carbapenem and beta-lactam resistance (Moya et al., 2010; Cabot et al., 2014; Wi et al., 2018), and making it as one of the most reliable options for the treatment of a broader spectrum of Non-CP-CRPA isolates. This is in line with the C/T susceptibility rate observed among CZA resistant isolates (69.8%), which is opposite to the CZA susceptibility rate observed in non-susceptible C/T isolates (11.1%), evidencing that C/T retains a good activity even in those isolates with a CZA resistant phenotype. Notwithstanding, since this study was fundamentally focused on the role that RND efflux pumps can play in baseline susceptibility to CZA and C/T, we do not present direct evidence about how other beta-lactam resistance mechanisms could be decreasing the activity to these new combined drugs and further studies are currently ongoing in our laboratory to clarify and deepen this hypothesis.

## Conclusion

One of the main conclusions derived from this study is that even though CZA and C/T presented an excellent rate of activity against Non-CP-CRPA clinical isolates, the activity of C/T was always higher than CZA, especially in the strains that showed an XDR and DTR phenotype. Furthermore, in this study, we propose the use of “baseline susceptibility” to extrapolate the concept of intrinsic resistance, usually defined in reference strains, to studies based on clinical isolates, regardless of whether they have been previously exposed to that or any other antimicrobial agent. Finally, our results show evidence that RND efflux systems play a role in the baseline susceptibility to CZA, C/T, or both, affecting the overall C/T susceptibility to a greater extent than CZA. Therefore, it is essential to keep exploring the discovery and development of efflux pump inhibitors that could be potentially applicable in clinical treatments.

## Data Availability

The original contributions presented in the study are included in the article/Supplementary Material, further inquiries can be directed to the corresponding authors.
